# Identification and Characterization of HSP90 Gene Family Reveals Involvement of HSP90, GRP94 and Not TRAP1 in Heat Stress Response in *Chlamys farreri*

**DOI:** 10.3390/genes12101592

**Published:** 2021-10-09

**Authors:** Haitao Yu, Zujing Yang, Mingyi Sui, Chang Cui, Yuqing Hu, Xiujiang Hou, Qiang Xing, Xiaoting Huang, Zhenmin Bao

**Affiliations:** 1 MOE Key Laboratory of Marine Genetics and Breeding, College of Marine Life Sciences, Ocean University of China, Qingdao 266003, China; haitao0532@foxmail.com (H.Y.); yzj@ouc.edu.cn (Z.Y.); suimingyi199808@163.com (M.S.); cuichang2019@139.com (C.C.); cnqdhyq@126.com (Y.H.); houxiujiang@stu.ouc.edu.cn (X.H.); qiangxing@ouc.edu.cn (Q.X.); zmbao@ouc.edu.cn (Z.B.); 2 Laboratory for Marine Fisheries Science and Food Production Processes, Pilot Qingdao National Laboratory for Marine Science and Technology, Qingdao 266071, China; 3 Laboratory of Tropical Marine Germplasm Resources and Breeding Engineering, SANYA Oceanographic Institution of the Ocean University of CHINA (SOI-OUC), Sanya 572000, China

**Keywords:** heat shock proteins 90, *Chlamys farreri*, heat stress, cell homeostasis

## Abstract

Heat shock proteins 90 (HSP90s) are a class of ubiquitous, highly conserved, and multi-functional molecular chaperones present in all living organisms. They assist protein folding processes to form functional proteins. In the present study, three *HSP90* genes, *CfHSP90*, *CfGRP94* and *CfTRAP1*, were successfully identified in the genome of *Chlamys farreri*. The length of *CfHSP90*, *CfGRP94* and *CfTRAP1* were 7211 bp, 26,457 bp, and 28,699 bp, each containing an open reading frame (ORF) of 2181 bp, 2397 bp, and 2181 bp, and encoding proteins of 726, 798, and 726 amino acids, respectively. A transcriptomic database demonstrated that *CfHSP90* and *CfGRP94* were the primary functional executors with high expression during larval development and in adult tissues, while *CfTRAP1* expression was low. Furthermore, all of the three *CfHSP90s* showed higher expression in gonads and ganglia as compared with other tissues, which indicated their probable involvement in gametogenesis and nerve signal transmission in *C. farreri*. In addition, under heat stress, the expressions of *CfHSP90* and *CfGRP94* were significantly up-regulated in the mantle, gill, and blood, but not in the heart. Nevertheless, the expression of *CfTRAP1* did not change significantly in the four tested tissues. Taken together, in coping with heat stress, *CfHSP90* and *CfGRP94* could help correct protein folding or salvage damaged proteins for cell homeostasis in *C. farreri*. Collectively, a comprehensive analysis of *CfHSP90s* in *C. farreri* was conducted. The study indicates the functional diversity of *CfHSP90s* in growth, development, and environmental response, and our findings may have implications for the subsequent in-depth exploration of *HSP90s* in invertebrates.

## 1. Introduction

Aquatic animals live in the complexity and variability of the marine environment and often experience a variety of environmental stresses, including temperature fluctuations, salinity shifts, oxygen deprivations, and pollution, which lead to a reduction in production and cause significant economic losses in marine aquaculture [1,2]. In recent years, unabated global warming has consequently increased the number of extremely high-temperature weather events [3]. Stress due to sudden changes in temperature or chronic heat stimuli above optimal levels can interrupt cellular homeostasis and result in serious growth and development deficiency in, and large-scale death of bivalves [4,5,6,7,8]. To date, a large number of organisms, especially those in the tropics, (such as insects, fish, reptiles, and amphibians) are living quite close to their thermal limits [9,10,11,12]. Therefore, the identification of the genes related to thermal responses is important to understand the molecular mechanisms underlying stress acclimatization.

Heat shock proteins (HSPs) are a family of molecular chaperones and were first discovered in 1962 in *Drosophila melanogaster* reared under heat stress conditions [13]. According to their monomeric molecular mass, HSPs can be broadly categorized into five major families: HSP100, HSP90, HSP70, HSP60, and the small HSP family [14]. HSPs play critical roles in the maintenance of protein homeostasis and protect organisms from environmental induced cellular damage [15]. When animals are exposed to continuous thermal stress, HSPs exert protective effects against the environmental perturbations [16]. Many insect species are seasonally exposed to suboptimal or supra-optimal temperatures which have led to the evolution of protective biochemical and physiological mechanisms, including the expression of HSPs [17]. In the Pacific oyster *Crassostrea gigas*, the expansion and massive upregulation of *HSP* genes may help the oyster’s adaption to sessile life in the highly stressful intertidal zone [18]. Under pressure, denatured proteins can be stabilized and folded by heat shock proteins. HSPs allow the binding proteins to either reach their natural conformations or target them for degradation and subsequent remove from the cell. This minimizes the probability of other proteins forming unproductive or cytotoxic aggregations [19]. 

In eukaryotes, every HSP families comprise multiple members and differs in their inducibilities, intracellular localization, and functions [20]. The members belonging to the HSP90 protein family are highly conserved and ubiquitous with an approximate molecular weight of 90-kDa. They are molecular chaperones that are importantly involved in the protein quality control (PQC) system and client-protein folding. Moreover, they can also regulate and assemble the protein complexes [21,22,23,24]. Additionally, HSP90s are essential for eukaryotic cell growth. They for a hub and interact with over 10% of the proteins in the proteome [25]. In mammalian cells, HSP90s are abundant and represent 1% to 3% of the total cytoplasmatic soluble proteins in physiological conditions [26]. The HSP90 family includes three main members: HSP90, located in the cytoplasm; GRP94 (94-kDa glucose-regulated protein), in the endoplasmic reticulum; and TRAP1 (tumor necrosis factor receptor-associated protein 1), primarily localized to the mitochondrial matrix and, to a certain extent, in the inter-membrane space [20]. There are two forms of HSP90 proteins in vertebrates, HSP90α (inducible) and HSP90β (constitutive) [15]. Unlike HSP90α, HSP90β lacks the glutamine-rich sequence (QTQDQ) at its N-terminus [27]. In invertebrates, only one form of HSP90 protein has been reported [28,29,30]. However, GRP94 and TRAP1 are found in both vertebrates and invertebrates [31]. Recently, many structural and functional similarities between GRP94 and HSP90 have been reported [32,33]. TRAP1, in recent years, has become a major therapeutic target for cancer and neurodegenerative disorders. It also plays a crucial role in the development of anti-viral and anti-protozoan treatment strategies [31]. As part of a large complex with other chaperones or essential cofactors, HSP90s can modify the misfolding of denatured proteins [20,34]. Moreover, they are also involved in hormonal signal transduction, cell differentiation, cell proliferation, apoptosis, morphogenesis, immune response, and stress defense in organisms [35,36]. Additionally, they play important roles in protecting organisms from stresses induced by a range of stressors, including heat or cold shock, hyperosmotic stress, food deprivation, reduced oxygen level, and heavy metals [37]. The induction of HSP90s under stress condition makes them biological monitors for environmental toxicants and stressors [37]. Previous studies indicate a positive relationship between thermotolerance and the transcript expression patterns of *HSP90s* in *D. melanogaster* [38]. In Pacific oysters, a short heat shock at a sublethal temperature can induce up-regulation of the expression of stress genes, including *HSP90s* [39]. After acute heat stress, the expression of *HSP90s* increases markedly in the scallops *Aropecten irradians* and *Patinopecten yessoensis* [40,41]. Taken together, HSP90s play important roles in heat stress responses and acclimatization of invertebrates. 

Zhikong scallop (*Chlamys farreri*), a commercially important species in China, has been cultivated since the 1970s. The large-scale death of scallops caused by high temperatures in summer seriously affects the development of the industry and causes serious economic losses to farmers [42]. The molecular mechanism underlying heat stress acclimatization has been poorly understood in this scallop. In the present study, we systematically identified and characterized the *HSP90* family in *C. farreri*, and examined the gene expression profiles during development stages, in healthy adult tissues, and under heat stress. The results may provide an important reference and contribute to a better understanding of the functioning of HSP90s and pave the way for their subsequent in-depth exploration of *HSP90s* in invertebrates.

## 2. Materials and Methods

### 2.1. Genome-Wide Identification and Sequence Analysis of HSP90 Genes in C. farreri 

The whole-genome database of *C. farreri* (PRJAN185456) [43] was used to query the typical HSP90 sequences of other species, including HSP90, GRP94, and TRAP1 in *Caenorhabditis elegans, Drosophila melanogaster, Crassostrea gigas, Homo sapiens, Mus musculus, Xenopus tropicalis,* and *Danio rerio* retrieved from NCBI (https://www.ncbi.nlm.nih.gov/guide/proteins/, accessed on 1 September 2021), Wormbase (https://wormbase.org/, accessed on 1 September 2021) and Flybase (http://flybase.org/, accessed on 1 September 2021) (Table S1). The amino acid sequences were predicted using ORF Finder (https://www.ncbi.nlm.nih.gov/orffinder/, accessed on 1 September 2021) and confirmed using BLASTP in the NCBI non-redundant protein sequence database. The conserved domains were predicted by SMART (http://smart.embl.de/, accessed on 1 September 2021) and the theoretical molecular mass and putative isoelectric point (pI) were predicted through the ProtParam tool (http://br.expasy.org/tools/protparam.html, accessed on 1 September 2021). 

### 2.2. Phylogenetic Analysis

For the identified HSP90 proteins sequences of *C. farreri* and other selected organisms, Multiple protein sequences alignments were performed using the ClustalW2 tool (http://www.ebi.ac.uk/Tools/msa/clustalo/, accessed on 1 September 2021). A phylogenetic tree was constructed using MEGA-7, based on the neighbor-joining method [44]. The robustness of the resulting phylogenies was tested by the reanalysis of 1000 bootstrap replicates.

### 2.3. Spatiotemporal Expression of HSP90 Genes in C. farreri

The expression profiles of *CfHSP90s* were analyzed using RNA-seq datasets of *C. farreri* (SRX2444844-SRX2444876, SRX2508197-SRX2508199, SRX2444668-SRX2444682, SRX2444950-SRX2444979, and SRX2445405-SRX2445440). The expression level was described by RPKM values (reads per kilobase per million mapped reads), which were obtained from the RNA-seq datasets, including different developmental stages (zygote, multicell, blastula, gastrula, trochophore, D-shaped larvae, early umbo, middle umbo, post umbo, eyespots larvae and juvenile), and adult tissues (striated muscle, smooth muscle, foot, mantle, eye, gill, blood, digestive gland, kidney, female gonad, male gonad, cerebral ganglia, and visceral ganglia). These RPKM values were log_10_ transformed and, subsequently, expression analysis by thermogram visualization was performed using the pheatmap package in R [45].

### 2.4. Expression Analysis of CfHSP90s under Heat Stress

The transcriptomic datasets of *C. farreri* in response to heat stress were independently constructed in our laboratory. A total of 160 scallops were randomly divided into four groups. The control group was kept in filtered and aerated seawater at 20 °C, the temperature of the sampling location. The other three groups were stress groups and kept in seawater at 27 °C, which was close to the maximum sea temperature in the *C. farreri* distributional area. Transcriptomic datasets at eight-time points (3 h, 6 h, 12 h, 24 h, 3 d, 6 d, 15 d, and 30 d) in four tissues, including mantle, gill, heart, and blood, and for three individuals per time point were used to analyze the expression levels of *CfHSP90s* under heat stress. The expression of *CfHSP90s* was calculated in TPM (transcripts per million) using the previously described formula [46]. Fold change (FC) for each test time point was calculated for the stress and control groups. Differentially expressed genes were identified and analyzed using the edgeR package with statistically significant cutoffs at |log_2_FC| > 1 and FDR < 0.05.

## 3. Results

### 3.1. Sequence Identification and Analysis

**Figure 1:**
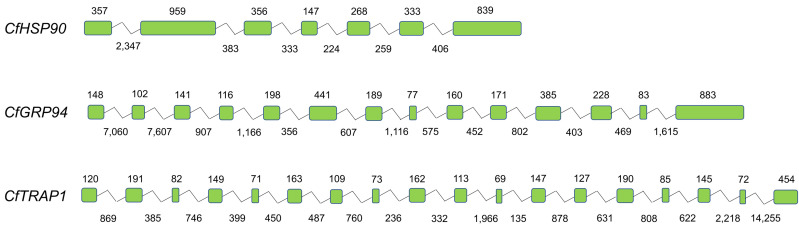
Gene structures for *CfHSP90s*. Green boxes indicate the exons, and the polylines indicate the introns. The numbers on the boxes indicate the lengths of the exons, the numbers under the lines indicate the lengths of introns.

**Figure 2:**
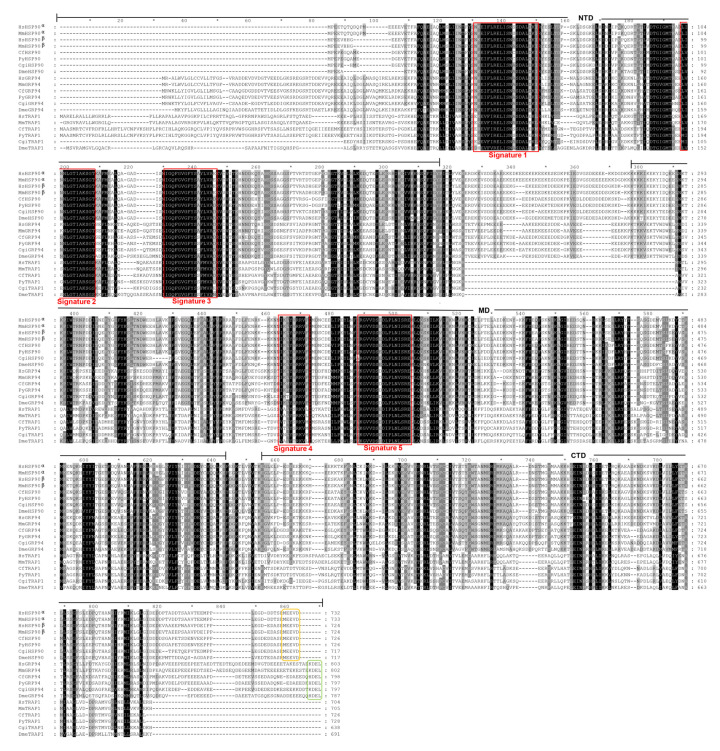
Multiple alignment of the amino acid sequences of HSP90s of *Chlamys farreri* with those of other species. Red box indicates HSP90 family signatures. The last five amino acids of HSP90 form the ‘MEEVD’ motif, which is marked with the orange box and the last four amino acids of GRP94 form the ‘H/KDEL’ motif which is marked with the green box.

Three *HSP90* family genes, *CfHSP90*, *CfGRP94*, and *CfTRAP1*, were identified in the genome of *C. farreri*, their presence was further confirmed using protein sequences of HSP90s. As shown in Table 1, the lengths of *CfHSP90*, *CfGRP94*, and *CfTRAP1* were 7211 bp, 26,457 bp, and 28,699 bp; open reading frame (ORF) consisted of 2181 bp, 2397 bp, and 2181 bp, which encoded proteins of 726, 798, and 726 amino acids, respectively. The predicted molecular weights ranged from 83.27 to 91.12 kDa, and theoretical pIs from 4.72 to 5.84. *CfHSP90* is composed of 7 exons, *CfGRP94* has 14 exons, and 18 exons were found in *CfTRAP1* (Figure 1). The amino acid sequences of CfHSP90 genes were aligned with those of the known HSP90s from other species (Figure 2). Although their genomic structures varied, three highly conserved domains, including the N-terminal domain (NTD), middle-domain (MD), and C-terminal domain (CTD), were found in amino acids encoded by all CfHSP90 family of genes (Figure 2). Specifically, there were three highly conserved family signatures in NTD, including signature 1 (NKEIFLRELISNSSDALDKIR), signature 2 (LGTIAKSGT), and signature 3 (IGQFGVGFYSAYLVAD). Two other family signatures were found in MD, including signature 4 (IKLYVRRVFI) and signature 5 (GIVDSEDLPLNISRE). Moreover, in HSP90, the last five amino acids form the ‘MEEVD’ motif at the CTD, but in GRP94, the CTD signal sequence was ‘H/KDEL’.

### 3.2. Phylogenetic Analysis

**Figure 3:**
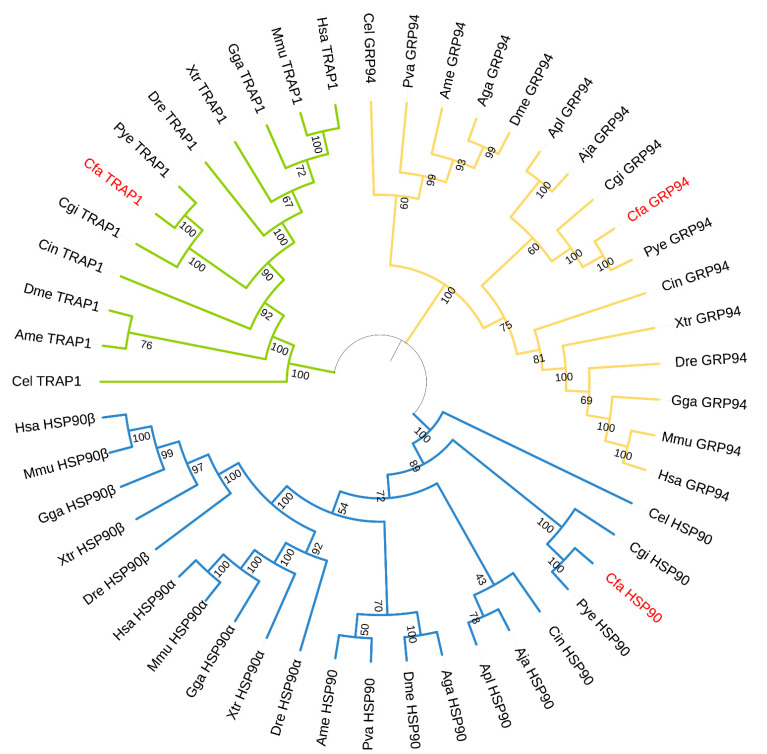
Phylogenetic tree of CfHSP90, CfGRP94, and CfTRAP1 with other HSP90s. The blue branches represent the HSP90 clade, the yellow branches represent the GRP94 clade, and the green branches represent the TRAP1 clade. CfHSP90 family is marked in red. The numbers under the tree branches indicate the bootstrap values from 1000 replicates.

A phylogenetic tree was constructed using the HSP90 protein sequences form *C. farreri* and other select organisms belonging to Nematoda, Arthropoda, Mollusca, Echinodermata, and Vertebrata (Figure 3). According to the phylogenetic analysis, the NJ tree is specifically clustered into three clades consisting of HSP90 proteins, GRP94 proteins, and TRAP1 proteins. In the HSP90 clade, CfHSP90 first clustered with HSP90 of another scallop species, *P. yessoensis*, followed by *C. gigas*, forming the branch of mollusks, and then clustered with *C. intestinalis+ A. japonicas+ A. planci,* and vertebrates. As for the classification of CfGRP94 and CfTRAP1, the results were similar to that of mollusks’ HSP90 protein, which indicated consistency in the evolutionary status of the three HSP90 subfamilies. In the clade composed of GRP94 proteins, the first cluster included mollusks and Echinodermata, while the vertebrates were assembled in the second cluster; the third cluster consisted of nematode and Arthropoda. In the TRAP1 tree, mollusks and vertebrates were directly clustered.

### 3.3. Spatiotemporal Expression of CfHSP90s

**Figure 4:**
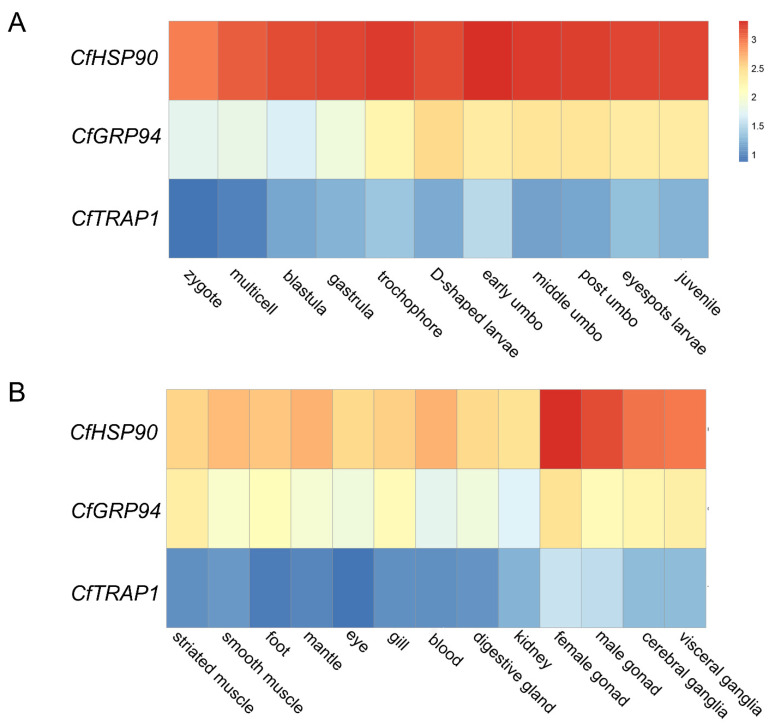
Expression analysis of *CfHSP90*, *CfGRP94*, and *CfTRAP1* at different developmental stages (**A**) and in adult tissues (**B**) of *C. farreri* based on the log_10_RPKM value.

The expression profiles of *CfHSP90s* at different developmental stages and, in adult tissues, were analyzed (Figure 4, Table S2). During the developmental processes (Figure 4A), the expression of *CfGRP94* was higher than that of *CfTRAP1* but lower than that of *CfHSP90*. In particular, the expression of *CfHSP90s* was constitutively high, with an average log_10_RPKM of 3.19. The expression of *CfGRP94* gradually increased from the zygote stage and reached its peak at the D-shaped larvae stage (log_10_RPKM = 2.52). Expression was moderate until the juvenile stage, with log_10_RPKM ranging from 2.35 to 2.43. In adult tissues (Figure 4B), the expression of *CfHSP90s* was ubiquitous, whereas the expression of *CfTRAP1* (average log_10_RPKM = 0.94) was substantially lower than both *CfHSP90* (average log_10_RPKM = 2.80) and *CfGRP94* (average log_10_RPKM = 2.01) expressions; similar expression patterns were observed in developmental stages. Specifically, *CfHSP90* and *CfGRP94* showed higher expression in the gonads (average log_10_RPKM = 3.37/2.28) and ganglia (average log_10_RPKM = 3.07/2.24) as compared with the other tissues.

### 3.4. Expression Profiles of CfHSP90s under Heat Stress

**Figure 5:**
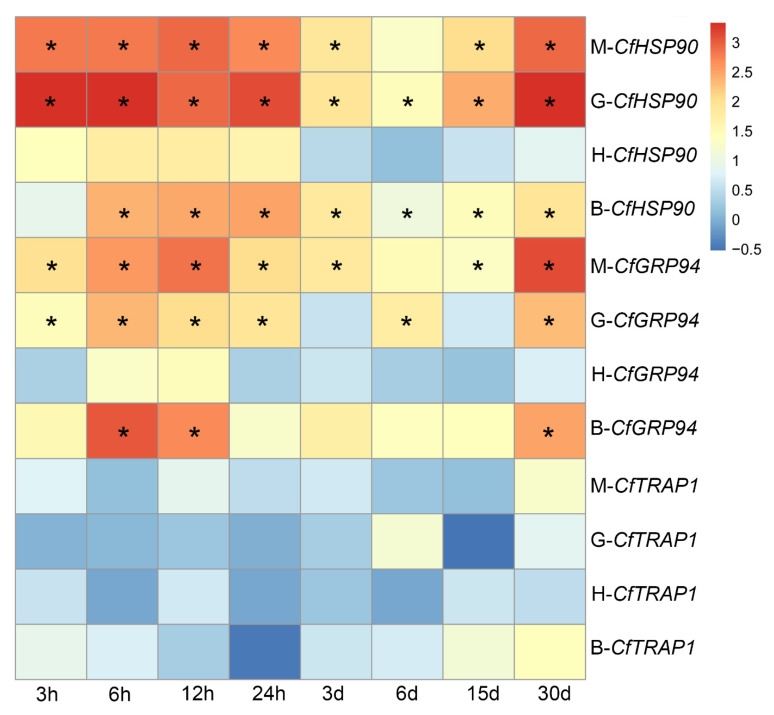
Expressions of *CfHSP90*, *CfGRP94*, and *CfTRAP1* in the mantle (M), gills (G), heart (H), and blood (B) of *C. farreri* under heat stress. The expressions of *CfHSP90s* at 0 h are used as controls. Values marked with asterisks indicate statistically significant differences compared with control expression (* |log_2_FC| > 1 and *FDR* < 0.05).

RNA-seq data (Table S2) showed diverse expression patterns of *CfHSP90s* under heat stress in the mantle, gill, heart, and blood of *C. farreri* (Figure 5). In general, the expression patterns of *CfHSP90* and *CfGRP94* were similar in the above-mentioned four tissue types. They were up-regulated under heat stress, whereas the expression of *CfTRAP1* did not change significantly under heat stress. Specifically, the expression of *CfHSP90* was significantly up-regulated under heat stress in the gill (at all time points), mantle (at all time points except 6 d), and blood (at all time points except 3 h). The expression of *CfGRP94* was significantly up-regulated under heat stress in the mantle (at all time points except 6 d), gill (at all time points except 3 d and 15 d), and blood (at 6 h, 12 h, and 30 d). In heart, the expressions of *CfHSP90* and *CfGRP94* did not change significantly. 

## 4. Discussion

The HSP90 family of genes has been identified in almost all the studied eukaryotic species. To data, the necessary roles of HSP90s in invertebrates have been investigated in response to biotic and abiotic stresses [28,37,47,48,49]. Based on the whole genome and transcriptome databases for *C. farreri*, we systematically identified and performed evolutionary analysis for *CfHSP90s*. We also investigated the expression profiles of these genes during larval development stages, in adult tissues, and under heat stress. 

A total of three *HSP90 genes* were identified in the genome of *C. farreri*. The number of *HSP90s* was the same as in other invertebrate species, such as *Caenorhabditis elegans* [50] and *C. gigas* [48]. But in the vertebrates, such as *Homo sapiens*, *Mus musculus*, four *HSP90s* are reported, including two *HSP90* isoforms (*HSP90α*, *HSP90β)*, *GRP94*, and *TRAP1* [20]. All proteins of the CfHSP90 gene family consist of three conserved domains, including the NTD, CTD, and MD. This was consistent with a previous report on other species [15]. Each domain within the HSP90 gene structure performed a specific function. For instance, ATP binds to NTD; proteins bind to MD, and the CTD is responsible for protein dimerization and consists of special motifs [20]. Specifically, the observed special motifs were the same as in other species, including the “MEEVD” motif in CfHSP90 and the “H/KDEL” motif in CfGRP94. Therefore, we speculated that CfHSPs may be executive of similar functions as those of HSPs in other species, such as to promote the folding of incorrectly folded proteins [20], and to activate steroid receptors [31]. Moreover, the NJ phylogenetic tree contained both orthologs and paralogs of the HSP90 family from vertebrates and invertebrates, which suggests that *HSP90* genes of *C. farreri* and vertebrates are potentially descended from a common ancestor. Additionally, there were three HSP90 family members in *C. farreri* and other invertebrate species, which indicated the maintenance of a relatively constant number; no large gene expansion was observed. However, both HSP90α and HSP90β exist only in vertebrates and are clustered separately and then clustered along with other HSP90s of invertebrates. This indicated that the two homologous isoforms of *HSP90* genes in vertebrates originated from an ancestral gene during evolution.

The *CfHSP90* genes were expressed at all developmental stages in *C. farreri*, which indicated that they played a significant role in the growth and development of the scallop larvae. The expressions of *CfHSP90* and *CfGRP94* were higher than that of *CfTRAP1*, which indicated *CfHSP90* and *CfGRP94* were functionally the main *HSP90s* in *C. farreri.* At early developmental stages, some transcripts of *CfHSP90* were detected in the zygote and multicell stages, which indicated the maternal expression of *CfHSP90*. Moreover, the continually high expression of *CfHSP90* in the developmental stages indicated its involvement in the regulation of growth and development of the scallop larvae, as the synthesis of large amounts of protein are required for cell division, cell differentiation, and organogenesis. Knorr and Vilcinskas silenced *HSP90* expression by RNAi in *Tribolium castaneum*. They found lethality in larvae within 10 days at all developmental stages [51]. Their results were in line with our findings. During early developmental stages, relatively higher levels of *CfGRP94* from the trochophore to juvenile stages indicated that the transcript initiated autologous synthesis functions. Thereafter, the expression dramatically increased in the D-shaped larva stage, crucial for the promotion of morphological and behavioral characteristics, formation of organs and shells, and the initiation of predation [52]. Similar expression profiles were also reported in *P. yessoensis* [40] and *M. musculus* [53,54]. During mouse embryonic development, *GRP94* transcripts are expressed in early embryos, while high levels of GRP94 protein are detected at later stages of organogenesis [55]. For *TRAP1*, to date, numerous studies have focused on understanding the relationship between its aberrant expression and tumorigenesis. Zhang et al. [56] report the upregulation of *TRAP1* expression in various human malignancies. Its aberrant expression may also lead to the development of cancer [57,58,59,60]. In our study, the expression of *CfTRAP1* was low and stable during developmental processes, in adult tissues, and under heat stress. However, the function of *CfTRAP1* remains ambiguous and needs further experimental evidence.

In adult tissues, *CfHSP90*, *CfGRP94*, and *CfTRAP1* were ubiquitously expressed, but their expression levels were different. Specifically, *CfHSP90s* were highly expressed in gonads of *C. farreri*, consistent with the findings for the homolog transcripts in *C. hongkongensis,* the black tiger shrimp, *Penaeus monodon*, marine crab, *Portunus trituberculatus*, and *Paphia undulata* [61,62,63]. *HSP90* usually activates the mitogen-activated protein kinase pathway, necessary for oocyte maturation in *Xenopus* [64]. During spermatogenesis, *HSP90* expression is largely up-regulated in rat testis [65]. Therefore, we speculated that *CfHSP90* was probably involved in the gametogenesis of *C. farreri.* Furthermore, *CfHSP90s* were also significantly expressed in the ganglia. In rabbits and bovine, the highest expressions of *HSP90s* are reported in the brain and they facilitate the binding of a glucocorticoid to its receptor [66,67]. Taken together, these results indicated that *CfHSP90s* could play an important role in nerve signal transmission in *C. farreri*. Similar results were also reported in *Macrobrachium nipponense*, in which *HSP90* is expressed ubiquitously in ganglia, heart, muscle, intestine, hemocytes, and gill; the highest expression is reported in the thoracic ganglia [68]. 

Temperature is an important abiotic factor that affects the organism’s survival, growth, and reproduction [69]. Previous studies have shown that when animals are exposed to continuous thermal stress, heat shock proteins (HSPs) exert protective effects [16]. Peng et al. [70] report that, during heat stress, the expression of *HSP70* and *HSP90* increase gradually to maximum levels, at 28 °C, in *Huso dauricus*. In *C. nobilis* [30], *Laternula elliptica* [71], *Argopecten irradians* [41], and *Sitodiplosis mosellana* [16], the expression of *HSP90* significantly increases during the thermal stress period. In our study, the expressions of *CfHSP90* and *CfGRP94* were significantly up-regulated in the mantle, gill, and blood under heat stress. We speculated that heat stress, at 27 °C, induced the expression of *CfHSP90s* to promote correct protein folding or the salvaging of damaged proteins for cell homeostasis. This is proven in *C. gigas* [72], *Paphia undulata* [63], and *Huso dauricus* [70]. Zhu et al. [72] report that heat stress in oysters destroys cellular homeostasis by damaging proteins, which further induces a highly conserved program of gene expression, leading to the selective transcription and translation of HSPs. In addition, we found that *CfHSP90* and *CfGRP94* had lower expression levels in the heart as compared with the mantle and gills. Their expressions were not different in the heart, which indicates that the roles of *CfHSP90* and *CfGRP94* in the heart were not significant.

## 5. Conclusions

In conclusion, we identified a complete *HSP90* family of genes, including *CfHSP90*, *CfGRP94*, and *CfTRAP1*, for the first time in the scallop *C. farreri.* The expression profiles of these genes were analyzed in the larval developmental stages, in adult tissues, and under heat stress. *CfHSP90* and *CfGRP94* were the main functional *HSP90s* for growth and development; these were expressed in almost all tissues. Under heat stress, the expressions of *CfHSP90* and *CfGRP94* were significantly up-regulated in the mantle, gill and blood, which suggested their crucial roles for coping with heat stress in *C. farreri*. The findings of this study provided a detailed explanation for *CfHSP90s* which could be implicated in the functions of *HSP90s* and further the understanding of the mechanism of environmental acclimatization in bivalves. 

## Figures and Tables

**Table 1:** Characteristics of *HSP90s* in *C. farreri*.

	HSP90	GRP94	TRAP1
Total length (bp)	7211	26,457	28,699
5’UTR length (bp)	357	96	—
3’UTR length (bp)	726	841	358
ORF length (bp)	2181	2397	2181
Amino acids length	726	798	726
Weight (kDa)	83.274	91.120	82.862
Theoretical pI	4.80	4.72	5.84
Number of exons	7	14	18
Number of introns	6	13	17
Number of alpha helixes	33	34	39
Number of beta strands	33	26	38
Number of coils	38	45	50
Number of turns	28	34	37

## Supplementary Materials

The following are available online at https://www.mdpi.com/article/10.3390/genes12101592/s1, Table S1: HSP90 protein sequences used in this study, Table S2: Expression profiles of CfHSP90s.

## References

[1] Park K., Lee J.S., Kang J.C., Kim J.W., Kwak I.S. (2015). Cascading effects from survival to physiological activities, and gene expression of heat shock protein 90 on the abalone *Haliotis discus* hannai responding to continuous thermal stress. Fish Shellfish. Immunol..

[2] Song L., Wang L., Qiu L., Zhang H. (2010). Bivalve immunity. Invertebrate Immunity.

[3] Masson-Delmotte V., Zhai P., Pörtner H.O., Roberts D., Skea J., Shukla P.R., Pirani A., Moufouma-Okia W., Péan C., Pidcock R. (2018). Global Warming of 1.5 °C.

[4] Liu S., Jiang X., Hu X., Gong J., Hwang H., Mai K. (2004). Effects of temperature on non-specific immune parameters in two scallop species: *Argopecten irradians* (Lamarck 1819) and *Chlamys farreri* (Jones & Preston 1904). Aquac. Res..

[5] Xiao J., Ford S.E., Yang H., Zhang G., Zhang F., Guo X. (2005). Studies on mass summer mortality of cultured zhikong scallops (*Chlamys farreri* Jones et Preston) in China. Aquaculture.

[6] Hornstein J., Espinosa E.P., Cerrato R.M., Lwiza K.M., Allam B. (2018). The influence of temperature stress on the physiology of the *Atlantic surfclam*, *Spisula solidissima*. Comp. Biochem. Physiol. Part A Mol. Integr. Physiol..

[7] Rahman M., Henderson S., Miller-Ezzy P., Li X., Qin J. (2019). Immune response to temperature stress in three bivalve species: Pacific oyster *Crassostrea gigas*, Mediterranean mussel *Mytilus galloprovincialis* and mud cockle *Katelysia rhytiphora*. Fish Shellfish. Immunol..

[8] Zhang W.-Y., Storey K.B., Dong Y.-W. (2020). Adaptations to the mudflat: Insights from physiological and transcriptional responses to thermal stress in a burrowing bivalve *Sinonovacula constricta*. Sci. Total. Environ..

[9] Stillman J.H. (2003). Acclimation capacity underlies susceptibility to climate change. Science.

[10] Deutsch C.A., Tewksbury J.J., Huey R.B., Sheldon K.S., Ghalambor C.K., Haak D.C., Martin P.R. (2008). Impacts of climate warming on terrestrial ectotherms across latitude. Proc. Natl. Acad. Sci. USA.

[11] Tewksbury J.J., Huey R.B., Deutsch C.A. (2008). Putting the heat on tropical animals. Science.

[12] Duarte H., Tejedo M., Katzenberger M., Marangoni F., Baldo D., Beltrán J.F., Martí D.A., Richter-Boix A., Gonzalez-Voyer A. (2012). Can amphibians take the heat? Vulnerability to climate warming in subtropical and temperate larval amphibian communities. Glob. Chang. Biol..

[13] Ritossa F. (1962). A new puffing pattern induced by temperature shock and DNP in Drosophila. Experientia.

[14] Joly A.-L., Wettstein G., Mignot G., Ghiringhelli F., Garrido C. (2010). Dual role of heat shock proteins as regulators of apoptosis and innate immunity. J. Innate Immun..

[15] Buchner J., Li J. (2013). Structure, function and regulation of the hsp90 machinery. Biomed. J..

[16] Cheng W., Li D., Wang Y., Liu Y., Zhu-Salzman K. (2016). Cloning of heat shock protein genes (hsp70, hsc70 and hsp90) and their expression in response to larval diapause and thermal stress in the wheat blossom midge, Sitodiplosis mosellana. J. Insect Physiol..

[17] Colinet H., Lee S.F., Hoffmann A. (2010). Temporal expression of heat shock genes during cold stress and recovery from chill coma in adult *Drosophila melanogaster*. FEBS J..

[18] Zhang G., Fang X., Guo X., Li L., Luo R., Xu F., Yang P., Zhang L., Wang X., Qi H. (2012). The oyster genome reveals stress adaptation and complexity of shell formation. Nature.

[19] Feder M.E., Hofmann G.E. (1999). Heat-shock proteins, molecular chaperones, and the stress response: Evolutionary and ecological physiology. Annu. Rev. Physiol..

[20] Hoter A., El-Sabban M.E., Naim H.Y. (2018). The HSP90 family: Structure, regulation, function, and implications in health and disease. Int. J. Mol. Sci..

[21] Borges J.C., Ramos C.H. (2005). Protein folding assisted by chaperones. Protein Pept. Lett..

[22] Pratt W.B., Toft D.O. (2003). Regulation of signaling protein function and trafficking by the hsp90/hsp70-based chaperone machinery. Exp. Biol. Med..

[23] Makhnevych T., Houry W.A. (2012). The role of Hsp90 in protein complex assembly. Biochim. Et Biophys. Acta (BBA)-Mol. Cell Res..

[24] Wandinger S.K., Richter K., Buchner J. (2008). The Hsp90 chaperone machinery. J. Biol. Chem..

[25] Zhao R., Houry W.A. (2007). Molecular interaction network of the Hsp90 chaperone system. Molecular Aspects of the Stress Response: Chaperones, Membranes and Networks.

[26] Lai B., Chin N., Stanek A., Keh W., Lanks K. (1984). Quantitation and intracellular localization of the 85K heat shock protein by using monoclonal and polyclonal antibodies. Mol. Cell. Biol..

[27] Lees-Miller S.P., Anderson C.W. (1989). The human double-stranded DNA-activated protein kinase phosphorylates the 90-kDa heat-shock protein, hsp90α at two NH2-terminal threonine residues. J. Biol. Chem..

[28] Liu T., Pan L., Cai Y., Miao J.J.G. (2015). Molecular cloning and sequence analysis of heat shock proteins 70 (HSP70) and 90 (HSP90) and their expression analysis when exposed to benzo (a) pyrene in the clam *Ruditapes philippinarum*. Gene.

[29] Wang Q., Wang J., Wang G., Wu C., Li J. (2017). Molecular cloning, sequencing and expression profiles of heat shock protein 90 (HSP90) in *Hyriopsis cumingii* exposed to different stressors: Temperature, cadmium and *Aeromonas hydrophila*. Aquac. Fish..

[30] Cheng D., Liu H., Zhang H., Tan K., Ye T., Ma H., Li S., Zheng H. (2020). Effects of thermal stress on mortality and HSP90 expression levels in the noble scallops *Chlamys nobilis* with different total carotenoid content. Cell Stress Chaperones.

[31] Jackson S.E. (2012). Hsp90: Structure and function. Molecular Chaperones.

[32] Dollins D.E., Warren J.J., Immormino R.M., Gewirth D.T. (2007). Structures of GRP94-nucleotide complexes reveal mechanistic differences between the hsp90 chaperones. Mol. Cell.

[33] Frey S., Leskovar A., Reinstein J., Buchner J. (2007). The ATPase cycle of the endoplasmic chaperone Grp94. J. Biol. Chem..

[34] Johnson J.L., Brown C. (2009). Plasticity of the Hsp90 chaperone machine in divergent eukaryotic organisms. Cell Stress Chaperones.

[35] Helmbrecht K., Zeise E., Rensing L. (2000). Chaperones in cell cycle regulation and mitogenic signal transduction: A review. Cell Prolif..

[36] Queitsch C., Sangster T.A., Lindquist S. (2002). Hsp90 as a capacitor of phenotypic variation. Nature.

[37] Gao Q., Zhao J., Song L., Qiu L., Yu Y., Zhang H., Ni D. (2008). Molecular cloning, characterization and expression of heat shock protein 90 gene in the haemocytes of bay scallop *Argopecten irradians*. Fish Shellfish. Immunol..

[38] Boher F., Trefault N., Piulachs M.-D., Bellés X., Godoy-Herrera R., Bozinovic F. (2012). Biogeographic origin and thermal acclimation interact to determine survival and hsp90 expression in Drosophila species submitted to thermal stress. Comp. Biochem. Physiol. Part A Mol. Integr. Physiol..

[39] Jo P.G., An K.W., Park M.S., Youngchoi C. (2008). mRNA expression of HSP90 and SOD, and physiological responses to thermal and osmotic stress in the Pacific oyster, Crassostrea gigas. Molluscan Res..

[40] Wang S., Li X., Li T., Wang H., Zhang X., Lou J., Xing Q., Hu X., Bao Z. (2018). The GRP94 gene of Yesso scallop (*Patinopecten yessoensis*): Characterization and expression regulation in response to thermal and bacterial stresses. Fish Shellfish. Immunol..

[41] Yang C., Wang L., Liu C., Zhou Z., Zhao X., Song L. (2015). The polymorphisms in the promoter of HSP90 gene and their association with heat tolerance of bay scallop. Cell Stress Chaperones.

[42] Zhang F., Yang H. (1999). Analysis of the causes of mass mortality of farming *Chlamys farreri* in summer in coastal areas of Shandong, China. Mar. Sci..

[43] Li Y., Sun X., Hu X., Xun X., Zhang J., Guo X., Jiao W., Zhang L., Liu W., Wang J. (2017). Scallop genome reveals molecular adaptations to semi-sessile life and neurotoxins. Nat. Commun..

[44] Kumar S., Stecher G., Tamura K. (2016). MEGA7: Molecular evolutionary genetics analysis version 7.0 for bigger datasets. Mol. Biol. Evol..

[45] Kolde R., Kolde M.R. (2015). Package ‘Pheatmap’.

[46] Wagner G.P., Kin K., Lynch V.J. (2012). Measurement of mRNA abundance using RNA-seq data: RPKM measure is inconsistent among samples. Theory Biosci..

[47] Gao Q., Song L., Ni D., Wu L., Zhang H., Chang Y. (2007). cDNA cloning and mRNA expression of heat shock protein 90 gene in the haemocytes of Zhikong scallop *Chlamys farreri*. Comp. Biochem. Physiol. Part B Biochem. Mol. Biol..

[48] Choi Y.K., Jo P.G., Choi C.Y. (2008). Cadmium affects the expression of heat shock protein 90 and metallothionein mRNA in the *Pacific oyster*, *Crassostrea gigas*. Comp. Biochem. Physiol. Part C Toxicol. Pharmacol..

[49] Zhang W., Wu C., Mai K., Chen Q., Xu W. (2011). Molecular cloning, characterization and expression analysis of heat shock protein 90 from Pacific abalone, *Haliotis discus* hannai Ino in response to dietary selenium. Fish Shellfish. Immunol..

[50] Haslbeck V., Kaiser C.J., Richter K. (2012). Hsp90 in non-mammalian metazoan model systems. Biochim. Et Biophys. Acta (BBA)-Mol. Cell Res..

[51] Knorr E., Vilcinskas A. (2011). Post-embryonic functions of HSP90 in *Tribolium castaneum* include the regulation of compound eye development. Dev. Genes Exolution.

[52] Shumway S.E., Parsons G.J. (2016). Scallops: Biology, Ecology, Aquaculture, and Fisheries.

[53] Mao C., Wang M., Luo B., Wey S., Dong D., Wesselschmidt R., Rawlings S., Lee A.S. (2010). Targeted mutation of the mouse Grp94 gene disrupts development and perturbs endoplasmic reticulum stress signaling. PLoS ONE.

[54] Wanderling S., Simen B.B., Ostrovsky O., Ahmed N.T., Vogen S.M., Gidalevitz T., Argon Y. (2007). GRP94 is essential for mesoderm induction and muscle development because it regulates insulin-like growth factor secretion. Mol. Biol. Cell.

[55] Barnes J., Smoak I.W. (1997). Immunolocalization and heart levels of GRP94 in the mouse during post-implantation development. Anat. Embryol..

[56] Zhang B., Wang J., Huang Z., Wei P., Liu Y., Hao J., Zhao L., Zhang F., Tu Y., Wei T. (2015). Aberrantly upregulated TRAP1 is required for tumorigenesis of breast cancer. Oncotarget.

[57] Agorreta J., Hu J., Liu D., Delia D., Turley H., Ferguson D.J., Iborra F., Pajares M.J., Larrayoz M., Zudaire I. (2014). TRAP1 regulates proliferation, mitochondrial function, and has prognostic significance in NSCLC. Mol. Cancer Res..

[58] Leav I., Plescia J., Goel H.L., Li J., Jiang Z., Cohen R.J., Languino L.R., Altieri D.C. (2010). Cytoprotective mitochondrial chaperone TRAP-1 as a novel molecular target in localized and metastatic prostate cancer. Am. J. Pathol..

[59] Fang W., Li X., Jiang Q., Liu Z., Yang H., Wang S., Xie S., Liu Q., Liu T., Huang J. (2008). Transcriptional patterns, biomarkers and pathways characterizing nasopharyngeal carcinoma of Southern China. J. Transl. Med..

[60] Xie S., Wang X., Gan S., Tang X., Kang X., Zhu S. (2020). The Mitochondrial Chaperone TRAP1 as a Candidate Target of Oncotherapy. Front. Oncol..

[61] Jiang S., Qiu L., Zhou F., Huang J., Guo Y., Yang K. (2009). Molecular cloning and expression analysis of a heat shock protein (Hsp90) gene from black tiger shrimp (*Penaeus monodon*). Mol. Biol. Rep..

[62] Zhang X.-Y., Zhang M.-Z., Zheng C.-J., Liu J., Hu H.-J. (2009). Identification of two hsp90 genes from the marine crab, *Portunus trituberculatus* and their specific expression profiles under different environmental conditions. Comp. Biochem. Physiol. Part C Toxicol. Pharmacol..

[63] Lin X., Wu X., Liu X. (2018). Temperature stress response of heat shock protein 90 (Hsp90) in the clam *Paphia undulata*. Aquac. Fish..

[64] Fisher D., Mandart E., Doree M. (2000). Hsp90 is required for c-Mos activation and biphasic MAP kinase activation in Xenopus oocytes. EMBO J..

[65] Krawczyk Z., Szymik N., Wiśniewski J. (1987). Expression of hsp70-related gene in developing and degenerating rat testis. Mol. Biol. Rep..

[66] Vamvakopoulos N.O. (1993). Tissue-specific expression of heat shock proteins 70 and 90: Potential implication for differential sensitivity of tissues to glucocorticoids. Mol. Cell. Biol..

[67] Itoh H., Toyoshima I., Mizunuma H., Kobayashi R., Tashima Y. (1990). Three-step purification method and characterization of the bovine brain 90-kDa heat shock protein. Arch. Biochem. Biophys..

[68] Zhao W., Chen L., Qin J., Wu P., Zhang F., Li E., Tang B. (2011). MnHSP90 cDNA characterization and its expression during the ovary development in oriental river prawn, *Macrobrachium nipponense*. Mol. Biol. Rep..

[69] Dong Y., Dong S. (2008). Induced thermotolerance and expression of heat shock protein 70 in sea cucumber *Apostichopus japonicus*. Fish. Sci..

[70] Peng G., Zhao W., Shi Z., Chen H., Liu Y., Wei J., Gao F. (2016). Cloning HSP70 and HSP90 genes of kaluga (*Huso dauricus*) and the effects of temperature and salinity stress on their gene expression. Cell Stress Chaperones.

[71] Kim M., Ahn I.-Y., Kim H., Cheon J., Park H. (2009). Molecular characterization and induction of heat shock protein 90 in the Antarctic bivalve *Laternula elliptica*. Cell Stress Chaperones.

[72] Zhu Q., Zhang L., Li L., Que H., Zhang G. (2016). Expression characterization of stress genes under high and low temperature stresses in the Pacific oyster, *Crassostrea gigas*. Mar. Biotechnol..

