# Supplementary material for: Identification and Characterization of HSP90 Gene Family Reveals Involvement of HSP90, GRP94 and Not TRAP1 in Heat Stress Response in Chlamys farreri
Source: Genes (Basel). 2021 Oct 9;12(10):1592. doi: 10.3390/genes12101592 (PMC8535295; doi:10.3390/genes12101592)
Supplement: Supplementary file 1 [file genes-12-01592-s001.zip › Table S1.pdf]

Table S1. HSP90 protein sequences information used in this study.

Classification	Species	Gene	GenBank accession number
Nematode	*Caenorhabditis elegans*	HSP90	NP_506626.1
		GRP94	NP_001255537.1
		TRAP1	pir||T16767
Arthropoda	*Drosophila melanogaster*	HSP90	NP_001261362.1
		GRP94	NP_651601.1
		TRAP1	NP_477439.2
	Apis mellifera	HSP90	NP_001153536.1
		GRP94	XP_395614.3
		TRAP1	XP_623366.2
	Anopheles gambiae	HSP90	sp|Q7PT10.3|
		GRP94	XP_321706.5
	Penaeus vannamei	HSP90	ADU03767.1
		GRP94	ANJ04737.1
Mollusca	*Patinopecten yessoensis*	HSP90	scaffold9985.1
		GRP94	scaffold10651.53
		TRAP1	scaffold3229.31
	Chlamys farreri	HSP90	scaffold41779.13
		GRP94	scaffold10699.10
		TRAP1	scaffold58059.9
	Crassostrea gigas	HSP90	ABS18268.1
		GRP94	BAF63637.1
		TRAP1	XP_011418207.2
Echinodermata	*Apostichopus japonicas*	HSP90	PIK36497.1
		GRP94	PIK57253.1
	Acanthaster planci	HSP90	XP_022103652.1
		GRP94	XP_022100344.1
Urochorda	*Ciona intestinalis*	HSP90	XP_009858093.1
		GRP94	XP_009861771.1
		TRAP1	XP_002123045.1
Vertebrata	*Homo sapiens*	HSP90α	NP_005339.3
		HSP90β	NP_001258898.1
		GRP94	NP_003290.1
		TRAP1	NP_057376.2
	Mus musculus	HSP90α	NP_034610.1
		HSP90β	NP_001004082.3
		GRP94	NP_035761.1
		TRAP1	AAH06864.1
	Gallus gallus	HSP90α	NP_001103255.1
		HSP90β	NP_996842.1
		GRP94	AAK69350.1
		TRAP1	NP_001006175.1

Xenopus tropicalis	HSP90α	NP_001016282.1
	HSP90β	NP_001025655.1
	GRP94	NP_001039228.1
	TRAP1	XP_002932506.1
Danio rerio	HSP90α	Q90474.3
	HSP90β	NP_571385.2
	GRP94	NP_937853.1
	TRAP1	NP_001107097.1
